# Interesting Case of Diabetes

**Published:** 1864-05

**Authors:** James P. White


ART. II.—*Interesting Case of Diabetes*—BY PROF. JAMES P. WHITE.

BUFFALO, May 2d, 1864.

*Dr. Miner:*

Dear Sir:—The enclosed letter of Col. Potter shows results so satisfactory in arresting the progress of a most intractable disease, that I send it you for publication in your valuable Journal, if you deem it worthy of insertion. The Colonel describes in the language of a very intelligent layman the plan of treatment which, with such modifications as are required by different individuals I have adopted during the last eight or ten years. It is scarcely to be expected that all patients possess the requisite perseverance and self-denial to secure the success attained by the writer of this letter. According to my observation little can be accomplished by the physician in the treatment of diabetes without the intelligent coöperation of the patient.

When Col. Potter was referred to me for advice by Dr. Eddy of Lewiston, N. Y., he had lost more than sixty pounds in weight, and was rapidly emaciating. At the time of making the diagnosis and putting him upon the course of hygiene and treatment which he describes, he was passing from ten to fifteen lbs. of urine daily, of the specific gravity of 1035 to 1045. Nearly four years have now elapsed since the adoption of this course, with the return of much of the weight lost, and still greater improvement in strength and vigor. It should be remarked that in the mean time that he has entirely changed his pursuits, and from being occupied in-doors, in mercantile business, he is now upon a farm, passing much of his time in the open air. It will be perceived that the Colonel has had to wage a constant warfare with his disease, and in doing so, has made himself familiar with the means of testing his urine, and has really manifested great skill in adopting his diet and therapeutics to the indications thus afforded. Hoping the letter may be useful in supplying hints to the practitioner and encouragement to the afflicted patient, I remain

Truly yours, JAMES P. WHITE.

"SPRING BROOK FARM, March 17, 1864,

*James P. White, M. D.:*

Dear Sir:—You will doubtless recollect your desponding patient of December, 1860, and his promise to communicate further with you after his retiracy upon a farm in Michigan the next year. Well, here I am, at the present, enjoying pretty good health, and expecting to live longer with more satisfaction to myself and family than I should if I had never been afflicted, although whenever I depart from the regimen prescribed, my urine becomes heavy, sometimes as high as 0.30, but usually in settled weather from 0.10 to 20. My remarkable preservation having excited a good deal of attention, and having the satisfaction of putting a number on the right track who have applied to me by letter, I deem it a duty I owe to humanity as well as yourself, to now comply with my promise, which I will do by copying your prescription verbatim, with a receipt or rather formula for the bran bread, and a reference to Dr. Joseph Bell's article, (see *Braithwaite's Journal*, January, 1857, p. 112,) for the use of cod liver oil at a time I was losing flesh rapidly, my voice weak and husky, and my lungs evidently sympathizing with the disease, and a formula for rennet sausage which has been of great benefit:

Strychnia grs. ii.
Acetic acid gutt. xxx.
Citrate of iron ℥ ss.
Water ℥ viii.

S Take a teaspoonful 4 times daily.

December, 1860. J. P. W.

Among the vegetables admissible are cabbage, celery, lettuce, spinach, water cress. (I find *sour krout* the best preparation of cabbage, although I use it in any form, and what is remarkable, that before I was afflicted it was almost indigestible.)

Drink: water in moderation; broths tepid or cold; claret wine if fond of it, (this is deceitful;) carbonate of soda and water.

*Hygiene,*—The surface should be kept warm, and occasional warm baths, followed by high friction. Passive exercise in the open air, avoiding excessive fatigue; cheerful society and early hours.

The hygiene is very important. I took a warm towel bath, followed with high friction daily for the first year; good beef in any form, and occasionally beef's liver done better with me than eggs. My urine occasionally, when as high as 0.32, deposits a sharp red sand, and is followed by rheumatic pains. These are removed by wine of colchium until it moves the bowels.

*Formula for bran cakes.*—Take a sufficient quantity of wheat bran, (say a quart,) boil it in two successive waters for one-fourth of an hour, each time straining it through a sieve, then wash it well with cold water on the sieve, until the water runs off perfectly clear, squeeze the bran in a cloth as dry as you can, then spread it thinly on a dish and place in a slow oven; if put in at night let it remain until the morning, when, if perfectly dry and crisp, it will be fit for grinding. The bran thus prepared must be ground in a fine mill, (I use a first class coffee mill screwed up tight,) and sifted through a wire sieve of such fineness as to require the use of a fine brush; this is particularly necessary if the bowels are irritable. Take of this bran, so ground, 3 or 4 oz., 3 new laid eggs, 2 oz. butter, and about a half pint of milk, mix the eggs with the milk, warm the butter with the other portion and stir it in, adding nutmeg or ginger, or any other agreeable spice. Bake in patti-pans one-half hour, in a quick oven, or like the common pan-cake for the same length of time. Butter them freely, and use no other bread until the urine is much lighter.

*Formula for Peptious or Sausage.*—Take three calves' rennets, rinsed off only, chop them up fine and season as you would sausage, insert them then in small cases, and boil for fifteen minutes, and then smoke them a little and use about 1½ inches with every meal.

You will recollect that you prescribed pepsin, but supposed it impossible to obtain; I found it, but it did not compare with the sausage made as above. My impression is that the sausage made as above is the best pepsin ever made, and must be useful in all stomach difficulties.

The following is Prof. Hadley's analysis at the time of your prescription:

*Dr. White:* December 18, 1860.

Dear Sir:—The following is a summary of the results of my analysis of the urine of your Albion patient. The sugar was determined with great care and accuracy:

I—*Afternoon urine, December* 14.

Specific gravity at 60° F.......................................1039.6

Has an *acid* reaction.

Deposits *uric acid* on standing two or three days.

Contains *sugar* L [C 12, H 14, O 14] 9.55 grs. to 100 meas.

43½ grs. to fluid ounce.

696 grs, to the pint.

Total passed at this time about 14 ℥ fl.

II—*Urine passed in the morning.*

Contains no *albumen.*

Fine crystals of *uric acid* on sides of vial.

Has an *acid* reaction.

Specific gravity at 60° F.......................................1045

Contains sugar 10.546 grs. to the 100 meas.
47 grs. to the fluid ounce.
754 grs, to the pint.

The *per centage* of sugar is by no means small. I have forgotten what Mr. P. estimated the daily measure of the urine.

Very truly yours, GEORGE HADLEY.

I have thus, dear Doctor, in a plain way given you the process (for which I am mainly indebted to you) to hold diabetes in check. Hoping you will live long to do good to the afflicted, I will only add that it is *a life work* to contend with the disease, but if it is only properly directed and the patient has courage, perseverance with *determination to live,* he may live comfortable with no more aches and pains than falls to the lot of man after he passes fifty, and until he reaches his allotted time of three-score and ten.

Very gratefully yours,

C. S. POTTER,
Kalamazoo.

P. S.—Give my regards to Prof. Hadley, thanking him for the desirable information he gave me in analyzing my own urine.

Last season I mowed (with a machine of course) 110 acres of hay and cut 60 of wheat, under a broiling sun and stood it as well as any man I had. Heretofore I have always been employed in-doors and needed a covering to go a mile.

Wherever you can benefit humanity or inspire confidence you are at liberty to use the foregoing."

---

REMEDY FOR BOILS.—Dr. D. B. Hoffman, of San Diego, Cal., says (*San Francisco Medical Press,* January, 1864,) that "Tincture of iodine, *double* strength, of the formula given in the United States Dispensary, applied thoroughly to boils, bunions, and carbuncles, will cut short the suppurative stages more than one-half, as well as relieve the patient of all pain. All of the feverish symptoms, with alternate agues, chills, and unpleasant feelings in the same, that are met with in delicate females and other persons, are relieved almost entirely by the first application."

